# Letter to the Editor

**Published:** 2025-06-20

**Authors:** Paul D. Rockswold, Jill U. Riehl

**Affiliations:** Defense Centers for Public Health–Portsmouth, VA: Dr. Rockswold, Ms. Riehl

We read with interest the report *Correlation Between Mean Temperature and Incidence of Tickborne Diseases Among Active Duty Service Members in the Contiguous U.S., 2000–2023* by Denagamage and Mabila in the March 2025 issue of *MSMR* . ^1^

We were pleased to see the Department of Defense's data systems being used to derive information for public health purposes. We noticed 3 items, however, that we think warrant attention because of how they might affect the interpretation of results. They include the potential over-diagnosis of Lyme disease, the findings between temperature and tick-borne diseases (TBDs) that are inconsistent with literature, and calculations based upon degrees Celsius rather than a temperature ratio scale with a true 0.

The potential for Lyme disease over-diagnosis is insufficiently addressed. The methods are based on encounter diagnoses, which may be based on clinical appearance with or without laboratory results. It is common for clinicians to over-diagnose Lyme disease based on symptoms and clinical findings alone, since diagnosis based solely on clinical presentation is unreliable. Several tick-borne illnesses have similar symptoms. A reaction to any tick bite, infected or not, may result in a skin response that could be mis-diagnosed as erythema migrans, the distinctive bullseye rash typically associated with Lyme disease. Southern tick-associated rash illness (STARI) is a tick-borne disease also characterized by a rash very similar to erythema migrans of Lyme disease, though it occurs most often in the southern U.S., where Lyme disease is considered rare. ^2^ The main vector of STARI is *Amblyomma americanum* , an aggressive species and most abundant tick in the mid-Atlantic and Southeast. The report does not adequately address how over-diagnosis limits the interpretation of results.

The geographical distribution of Lyme disease and *Ixodes scapularis* activity peaks appear to be inadequately addressed. Active surveillance data in southern Virginia show that adult *I. scapularis* , the vector of the pathogen that causes Lyme disease, is most active in the Southeast in late winter and early fall (i.e., not hot summer months). ^3^ Historically, Lyme disease is more prevalent in the northern and midwestern U.S.; increased Lyme disease incidence in service members in the South is inconsistent with existing literature. Even though the tick vector is seen in the South, multiple factors account for its lower Lyme disease incidence. The more notable factors include that host-seeking or questing behavior differs between *I. scapularis* in the North compared to the South, resulting in dramatic differences in Lyme disease prevalence. In the Southeast, ticks tend to feed on reptiles more than mammals. Since reptiles are inefficient reservoirs for Lyme disease spirochetes, there is lower incidence of the spirochetes in reptiles and ticks. ^4^ Climate change may be responsible for habitat expansion but alone is insufficient to account for the changes in Lyme disease distribution.

Additionally, we had concerns about the impact of certain assumptions on the overall conclusions and statements regarding temperature. When using a ratio scale, “equality of ratios as well as equality of intervals may be determined. Fundamental to the ratio scale is a true zero point.” ^5^ For example, a 6-foot-tall adult is 2 times the height of a 3-foot-tall child, which matches the ratio of 2 between the values. If these 2 were to stand in a hole 2 feet deep, the top of the adult head would be 4 feet above ground level and the child's head would be 1 foot above ground level. Having them stand in a hole does not make the adult 4 times taller than the child.

Turning to the report, the text reports a 0.6°C increase (5.3%) in overall annual mean temperature from 2000 to 2023. We assume the starting temperature is 11.3°C, increasing to an end temperature of 11.9°C (starting and ending temperatures were not reported). The temperature scale affects the results, however. Consider the following, where the first bullet captures what was reported, the second uses Fahrenheit, and the third uses the Kelvin scale:

- Celsius: the range from 11.3°C to 11.9°C resulted in a 5.3% increase;
- Fahrenheit: the range from 52.3°F to 55.2°F would result in a 5.5% increase;
- Kelvin: the range from 284.5 K to 285.1 K would result in a 0.2% increase.

Referring to the example, using Celsius and Fahrenheit is like standing in a hole: It misrepresents the reality. A calculation like this must be based upon a ratio scale with a true 0, such as the Kelvin scale, where a change of 0 K is identical to a change of 1°C; also, 0 K equals -273.2°C and 0°C equals 273.2 K. This affects other areas of the report, as well. In the caption of **Figure 3a** , the 9.9% increase of annual mean temperature from 2011 to 2012 should be reported as about 0.4%. The 2.3% increase from 2015 to 2016 should be reported as about 0.1%.

Thank you for your consideration.
