# Supplementary material for: Letter to the Editor
Source: MSMR. 2025 Jun 20;32(6):25–6. (PMC12266730)
Supplement: Related texts

# In Reply:

We thank Dr. Rockswold and Ms. Riehl for their interest in our article, *Correlation Between Mean Temperature and Incidence of Tick-borne Diseases Among Active Duty Service Members in the Contiguous U.S., 2000–2023* , published in the March 2025 issue of *MSMR* .

We agree that Lyme disease may have been over-diagnosed in our study, particularly in areas where erythema migrans or similar rashes are prevalent. Following the Armed Forces Health Surveillance Division (AFHSD) Lyme disease case definition, our study sample includes unconfirmed cases from medical encounters as well as reportable medical events (RMEs) confirmed by laboratory data per the 2022 *Armed Forces RME Guidelines and Case Definitions* . ^1^ Despite potential over-reporting due to inclusion of unconfirmed cases from medical encounters, our study's large sample size and lengthy surveillance period allowed identification of temporal and regional patterns that can guide efforts to protect service members from Lyme disease. Future studies using exclusively laboratory data may provide more accurate incidence estimates.

We appreciate the note on how *Ixodes scapularis* behavior and host preferences affect Lyme disease distribution. As stated, Lyme disease is historically more prevalent in the northern and midwestern U.S., and our crude incidence rates reflect this pattern, as shown in **Table 1** (Northeast, 52.2 cases per 100,000 person-years; Upper Midwest, 13.3 cases per 100,000 person-years). Our adjusted incidence rate ratios (aIRRs) indicate, however, a relatively higher burden in the Southeast after controlling for climatic and demographic variables. We agree that this requires further investigation to discern if adjusting for temperature and precipitation measures alongside demographic variables accounts for such a discrepancy. Analysis of entomologic and ecological factors was beyond the scope of our study, but future research including these data may clarify how tick behavior, host preferences, and habitat influence regional variation in incidence of tick-borne diseases.

While Celsius is widely used in public health literature, we recognize that percentage changes based on a scale without a true 0, such as Celsius and Fahrenheit, can be misleading. Our intent in citing percentage increases was to contextualize changes in temperature over time, while reporting temperature in Celsius was intended to make it easier to compare our results with those of similar studies. We agree, however, that expressing changes in absolute degrees Celsius, or percent change using Kelvin, are more appropriate in this context.

## References

[B6] Armed Forces Health Surveillance Branch . Armed Forces Reportable Medical Events Guidelines and Case Definitions. Defense Health Agency, U.S. Dept. of Defense . Accessed May 22, 2025 . https://www.health.mil/reference-center/publications/2022/11/01/armed-forces-reportable-medical-events-guidelines
